# A spatial analysis of heat stress related emergency room visits in rural Southern Ontario during heat waves

**DOI:** 10.1186/s12873-015-0043-4

**Published:** 2015-08-06

**Authors:** Katherine E. Bishop-Williams, Olaf Berke, David L. Pearl, David F. Kelton

**Affiliations:** Department of Population Medicine, Ontario Veterinary College, University of Guelph, Guelph, ON N1G 2W1 Canada

## Abstract

**Background:**

In Southern Ontario, climate change may have given rise to an increasing occurrence of heat waves since the year 2000, which can cause heat stress to the general public, and potentially have detrimental health consequences. Heat waves are defined as three consecutive days with temperatures of 32 °C and above. Heat stress is the level of discomfort. A variety of heat stress indices have been proposed to measure heat stress (e.g., the heat stress index (HSI)), and has been shown to predict increases in morbidity and/or mortality rates in humans and other species. Maps visualizing the distribution of heat stress can provide information about related health risks and insight for control strategies. Information to inform heat wave preparedness models in Ontario was previously only available for major metropolitan areas.

**Methods:**

Hospitals in communities of fewer than 100,000 individuals were recruited for a pilot study by telephone. The number of people visiting the emergency room or 24-hour urgent care service was collected for a total of 27 days, covering three heat waves and six 3-day control periods from 2010–2012. The heat stress index was spatially predicted using data from 37 weather stations across Southern Ontario by geostatistical kriging. Poisson regression modeling was applied to determine the rate of increased number of emergency room visits in rural hospitals with respect to the HSI.

**Results:**

During a heat wave, the average rate of emergency room visits was 1.11 times higher than during a control period (IRR = 1.11, CI_95%_ (IRR) = (1.07,1.15), p ≤ 0.001). In a univariable model, HSI was not a significant predictor of emergency room visits, but when accounting for the confounding effect of a spatial trend polynomial in the hospital location coordinates, a one unit increase in HSI predicted an increase in daily emergency rooms visits by 0.4 % (IRR = 1.004, CI_95%_(IRR) = (1.0005,1.007), p = 0.024) across the region. One high-risk cluster and no low risk clusters were identified in the southwestern portion of the study area by the spatial scan statistic during heat waves. The high-risk cluster is located in a region with high levels of heat stress during heat waves.

**Conclusions:**

This finding will aid hospitals and rural public health units in preventing and preparing for emergencies of foreseeable heat waves. Future research is needed to assess the relation between heat stress and individual characteristics and demographics of rural communities in Ontario.

## Background

Heat stress is a known health risk in major metropolitan areas around the world, including Southern Ontario [1–4]. Heat stress is the physiological response to extreme heat which can result in discomfort, morbidity and mortality. During periods of extreme heat such as heat waves, the likelihood of heat stress is increased. Environment Canada [5] defines a heat wave as three consecutive days of maximum temperatures of 32 °C or higher. A heat stress index (HSI) can be used to quantify the level of discomfort an individual feels. A variety of HSIs have been developed, though in a comparison of these indices Barnett et al. [6] illustrate that none of them is superior to the others. Therefore, Barnett et al. [6] suggest choosing an HSI based on available data. Hartz et al. [7] found in a study of the Chicago area that HSI is a better indicator of increase in predicted emergency room visits and 9-1-1 calls than ambient temperature. In Korea, Na et al. [8] found that a temperature threshold was a better predictor of heat-related illness, where increases in the relative risk (RR) corresponded to increases in emergency room visits; however, their threshold of 31.2 °C (i.e. 88 °F) is slightly lower than the heat wave definition established by Environment Canada [5].

Heat waves are an ideal period to study heat stress in Southern Ontario because a heat wave is illustrative of the effects of prolonged exposure to extreme heat [9]. A study in Sweden found that excess mortality due to heat stress increased 2.0–3.9 % per day [9]. In Korea, estimates suggest that mortality increases 4.1 % during heat waves [10]. Many simulations and models predict the increase of frequency, intensity and duration of heat waves in the future [11, 12]. This increase will result in an increased public health burden for communities across Ontario, and likely throughout all of Canada. In Southern Ontario, there have been one or more heat waves each summer since 2003 [5].

While data are available to illustrate the current presence of heat waves in Southern Ontario, little is known about the impact these have on the population. Heat stress studies and their associated impacts are common for major metropolitan areas around the world such as Athens [13], Shanghai [14], Paris [15], Sydney [16], and Madrid [17]. Studies of heat stress in the neighbouring United States of America indicate the presence of heat wave threats in North American cities such as Chicago [18], Baltimore [19], and St. Louis and Kansas City [20]. Studies of Canadian metropolitan area heat stress and its impacts illustrate the real threat of heat waves in cities like Montreal [21] and Toronto [1–4].

In contrast to urban settings, studies in rural areas are scarce. However, Martinez Navarro et al. [22] report that the impact of heat waves on mortality could also be seen in rural communities in Spain.

Previous population based heat stress studies applied a variety of proxy measures to establish a link between heat waves and heat-related illness. Weather data have been collected by satellite imaging [1] or from nearby weather stations [23]. Although these measures can provide a detailed image and understanding of the spatial distribution of heat stress in an area, there are two major drawbacks to using satellite imaging for this study. Satellite images are generally only available 1–2 times per day, which may not include the daily maximum or minimum heat stress level for a region on a given day [24]. Furthermore, satellite imaging does not correlate to ambient temperature in rural communities as well as meteorological station measurements do, as a result of the amount of green space in the region [24]. Health data have also been collected by a variety of proxy measures. Various data sources have been investigated such as 9-1-1 dispatch data [1, 25], emergency room visits [20], death records [22], surveillance data [8, 26], or a combination of the above [16]. Increases in myocardial infarction and other circulatory disorders, respiratory disorders as well as falls and respective injuries are all known to be associated with extreme heat, which may be missed when only specific illness are considered [17]. Moreover, the risks of illness or injuries as a result of heat stress are compounded for those who are obese, or with pre-existing cardiovascular disorders, respiratory disease and diabetes mellitus [27]. These indirect results of heat stress would not be documented as heat-related illness, but may be an indication that stress is high in the community.

The media can have a substantial impact on the health outcomes of a community during a heat wave. In New York City, heat wave warning systems were analyzed and compared to see which system for reducing mortality due to heat waves was the most effective [28]. The researchers determined that systems which warn local residents of a heat wave based on the daily maximum temperature had the greatest ability to reduce mortality [28]. No such studies exist for rural communities at this time.

According to projections for future climate change, heat waves are anticipated to increase in frequency, intensity and duration [11, 12]. The public health burden associated with heat waves and heat stress is anticipated to increase as well [29]. Peng et al. [29] predicted that increases in extreme heat as a result of heat waves will cause excess mortality of 166–2,217 deaths per year by the year 2081 in Chicago. Moreover, Josseran et al. [30] found that the public health burden associated with specific ailments such as dehydration, hyperthermia, malaise, hyponatremia, renal colic and renal failure increase during heat wave events. To date, few studies have demonstrated if similar health issues arise in rural communities or if the magnitude of these relationships differs.

**Fig. 1:**
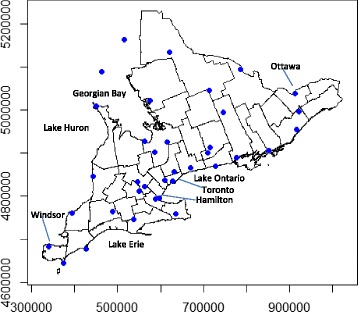
Location of meteorological stations in and around Southern Ontario which provide hourly interval data collection. Blue points represent the location of meteorological stations across Southern Ontario. Axes units are in meters

Heat stress distribution in Southern Ontario is stable over time [31], and thus is predictable during a heat wave. In general, heat stress is high in and around major metropolitan areas such as the Greater Toronto Area (GTA). In Southern Ontario, heat stress was also high in the southwestern region surrounding London and in the east near Ottawa (Fig. 1).

The goal of this study was to investigate whether heat waves pose a public health threat to residents of Southern Ontario using retrospective analysis of rural hospital emergency rooms visits during heat waves from 2010–2012. Specific objectives are: (i) to identify the levels of heat stress experienced by rural communities of Southern Ontario during a heat wave; (ii) to determine if heat waves have a significant impact on the number of visits to rural hospital emergency rooms; and (iii) to identify areas of high-risk for heat stress related illness in Southern Ontario.

## Methods

### Study area and population

Weather data for the region of Southern Ontario were collected in its entirety, defined by the public health units of Ontario’s definition of Southern Ontario. The study area is home to nearly one third of the Canadian population and borders the St. Lawrence River, Lake Ontario, Lake Erie, and the southern portions of Lake Huron and Georgian Bay. This study area is thus quite different from areas investigated in previous work because it is substantially larger than most preceding heat stress map regions. Communities were defined as rural if the population of the town for the hospital mailing address was fewer than 100,000 people. A total of 61 communities with hospitals qualified for the study. Of 50 hospitals with emergency rooms contacted, 24 hospitals provided data for the number of visits to the emergency room for pre-specified dates.

### Data collection

Heat waves were identified using a search of the popular press, which indicated possible heat wave dates. As a result of the variability in definitions for a heat wave, each heat wave was confirmed to meet the Environment Canada definition of a heat wave (3 days of temperatures of at least 32 °C) at a minimum of 3 weather station locations in Southern Ontario. Importantly, in Canada, heat waves are identified by their daily maximum temperatures, rather than mean temperatures. Canadian communities can experience summer maximum temperatures up to 40 °C, and winter minimum temperatures of −40 °C. As a result of these extremes, bodies are generally well adapted to moderate temperatures, and the daily extremes pose intense risk to the population in contrast to prolonged mean temperatures. Control periods were selected based on dates of confirmed heat waves to start 3 weeks (21 days) prior to the start of the heat wave and 3 weeks following the start of the heat wave. Control periods were matched for day of the week to control potential weekday effects.

Weather data for this study were previously collected as part of another study [31]. Briefly, weather data were collected from the Environment Canada database, the National Climate Data and Information Archive [5], from 37 hourly operating weather stations on 27 dates.

A list of hospitals in Southern Ontario was obtained from the Ontario Hospital Association [32]. Hospital addresses were extracted from this database and the population of the town was retrieved from Statistics Canada [33]. Hospitals were contacted by telephone between October 16th, 2013 and November 25th, 2013. A short script (1–2 min) was used to inform hospital contacts of the nature of the request. A written data request was made to the Health Records Departments of the hospitals, after contact had been established by phone. The raw number of visits to the ER for non-scheduled appointments during a three-day heat wave or control period was requested for a total of 9 periods (outlined in Table 1). Data collected from hospitals included the population of the community in which the hospital was located, number of emergency room visits per day and the method for record keeping at the hospital.

**Table 1:** Dates of exposure and control periods

Exposure Period	Control Period 1 (Preceding)	Control Period 2 (Following)
June 19–21, 2012	May 29–31, 2012	July 10–12, 2012
July 20–22, 2011	June 29–July 1, 2011	Aug 17–19, 2011
July 5–7, 2010	June 14–16, 2010	Aug 2–4, 2010

For this pilot study, emergency room visit numbers were collected without personal identifiers to determine if heat stress was an existing problem in rural communities in Southern Ontario. When using emergency room data, all visits to emergency rooms were compared between heat waves and control periods. Control weeks were determined as being 3 weeks (i.e. 21 days) prior to and following respective heat waves, matched for day of the week bias. To illustrate the increase in morbidity rates during periods of extreme heat exposure ER admissions for all reasons were assessed. The Research Ethics Board at the University of Guelph did not require ethics approval for this study.

### Mapping

The HSI defined by Johnson and Vanjonack [34] was used in this study:

$$ \mathrm{H}\mathrm{S}\mathrm{I} = {T}_{DB} + \left({T}_{DP}*\ 0.36\right) + 41.5 $$

where *T*_*DB*_ and *T*_*DP*_ denote dry bulb temperature and dew point temperature respectively. All temperatures are measured in degrees Celsius. Since all indicators for calculating the HSI are considered equally predictive over space [6], the HSI outlined above was chosen for data completeness. The HSI was calculated for each of the 37 weather stations as a 3-day average of the daily maxima. Geostatistical kriging [35] was used to predict the spatial pattern of heat stress for the entire study area, (i.e., Southern Ontario). The HSI value for each hospital during the heat wave and control periods was determined by applying the previously generated heat stress maps [31].

### Modelling and analysis

The average number of emergency room visits per day was calculated by the average of each of the three days of a heat wave or control to reflect the calculation of the HSI average over the 3-day period. Each period was dichotomized as a heat wave or control period for analysis.

A backwards stepwise procedure was used to build the Poisson regression model. A univariable analysis was done to screen for potentially significant variables at a liberal significance level α = 0.2. Northing, easting, and the dichotomized heat wave indicator were initially modeled. The least significant predictors were removed consecutively until all remaining parameters in the model were significant. Confounders were tested based on biological plausibility, and included in the final model if they changed the coefficient of a significant predictor by greater than 20 %. Appropriate diagnostics were used to determine the quality of the fit of the model. Goodness of fit was assessed using a quantile-quantile plot of Anscombe residuals.

All spatial analyses were performed in R computing software version 3.1.0 [36], using a significance value of α = 0.05 unless otherwise stated.

To test for areas of high or low risk, a spatial scan test was conducted for Southern Ontario to determine if areas were at increased risk of heat stress. The incidence rate ratio (IRR) was estimated in SaTScan [37] by inputting the number of emergency room visit records per hospital and the population of the community. Spatial coordinates in latitude and longitude were translated to Cartesian coordinates in Universal Transverse Mercator (UTM) 17 North for analysis. A purely spatial model (i.e. no temporal analysis) using the Poisson distribution was used to test for clusters of high or low incidence of emergency room visits. Since the distribution of heat stress in Southern Ontario was previously shown to be stable [31], all data for the 9 periods were used together to increase power for the scan test; and a space-time scan test was not applied. Analysis was conducted in SaTScan vs 9.3 [37] using a significance level of α = 0.05, 999 Monte Carlo permutations and a maximum scanning window of 50 % of the population. The p-value was estimated using the standard procedure in SaTScan.

## Results

### Descriptive results

Weather data were available from 37 weather stations across Southern Ontario, illustrated in Fig. 1. The HSI at each weather station was estimated for every hour of available data. The maximum daily HSI value occurred most frequently between 12:00 and 15:00 (58 %), and least often between 04:00 and 07:00 (0.4 %).

**Fig. 2:**
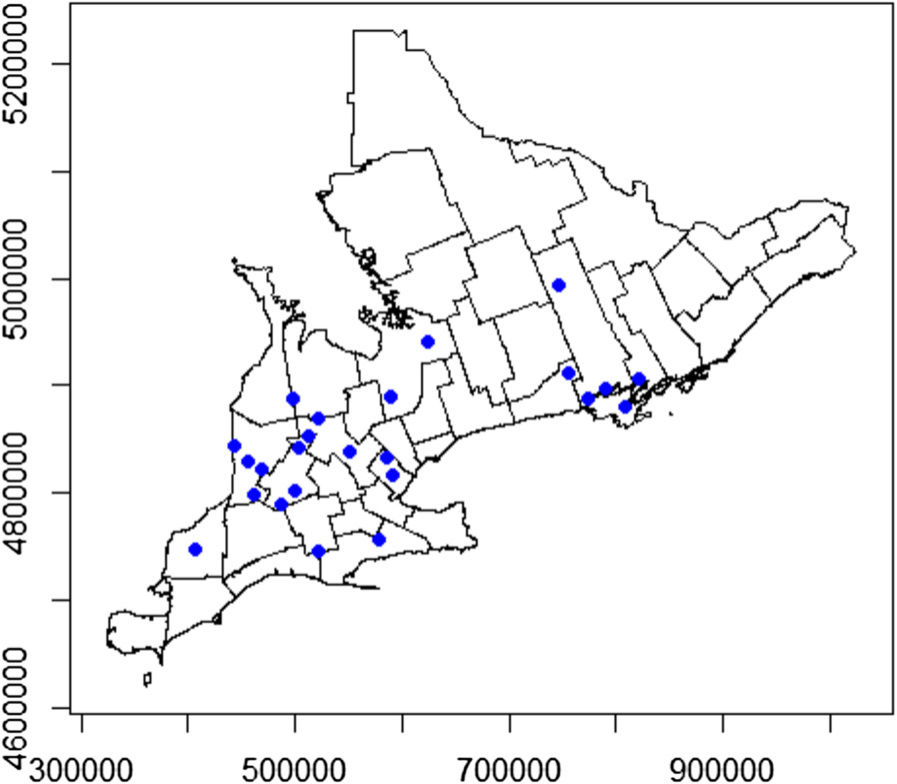
Location of 24 participating hospitals. Locations of 24 rural community hospitals in Southern Ontario which provided data for the number of emergency room visits per day for a study from 2010–2012 are marked with blue points. Axes units are in meters

The response rate for this study was 48 %. Of a total 50 rural community hospitals in Southern Ontario which are located in towns of fewer than 100,000 people and which had an emergency room or 24-hour urgent care centre, 24 hospitals were willing to participate. The average population of the towns from which these hospitals come is 16,324 people (range: 2,579–83,575). Hospitals that participated in the study were primarily located in southwestern Ontario and in the Thousand Islands area along the St. Lawrence River (see Fig. 2).

**Fig. 3:**
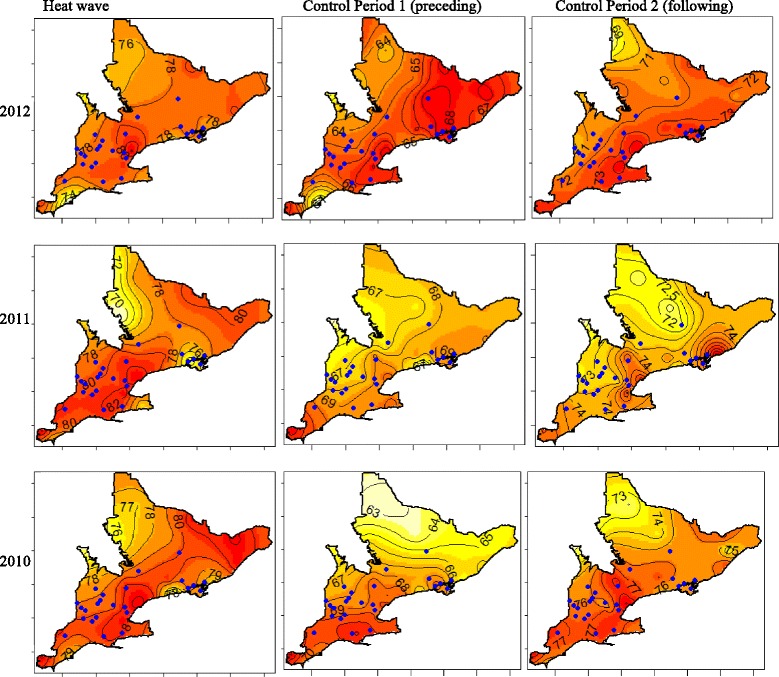
Heat stress maps for Southern Ontario during each of the 3 heat waves and 6 control periods. Heat stress is represented on Isopleth maps where darkest colours (red) represent the most intense heat stress levels. Light colours represent the areas of least intense heat stress. Contour lines indicate the level of HSI reported in heat stress units. Each of the 24 hospital locations are marked in blue

Heat stress maps were created previously for Southern Ontario [31]. Heat stress maps of the three heat waves and six control periods are depicted in Fig. 3. These heat stress maps also illustrate the location of the 24 hospitals that were enrolled in this study. Heat stress follows a predictable pattern over space and time during a heat wave in Southern Ontario. Heat stress is highest in the southern portion of the study region, particularly concentrated around the Greater Toronto Area, and in a belt stretching from Lake Ontario to Lake Huron. The average onsite HSI at the 24 hospitals during a heat wave was 79 (range: 76–82), and during a control period the average onsite HSI at the 24 hospitals was 71 units (range: 64–78). These measures, in addition to maps of the distribution of heat stress, can be used to describe the HSI intensity during the heat waves and respective control periods.

The average number of hospital visits to these emergency rooms during a control period was 61 emergency room admissions per day (range: 18–182). The average number of emergency room visits per day during a heat wave period was 63 (range: 21–174).

### Analytic results

Univariable Poisson regression modeling identified a spatial trend polynomial in the hospital location coordinates, and the binary heat wave indicator as significant predictors of emergency room visits in Southern Ontario (*p* < 0.20). When modeled on its own, i.e. in univariable analysis, the HSI was not a significant predictor of emergency room visits in Southern Ontario (*p* = 0.802).

The Poisson regression model was built using backwards stepwise modelling. A dichotomized heat wave variable, northing, and easting were significantly associated with the likelihood of having increased emergency room visits in univariable analysis. In the final model, the heat wave indicator variable was the only significantly associated parameter with emergency room visits. The Akaike Information Criterion (AIC) was used to assist in selecting the best model. The incidence rate of emergency room admissions was 1.11 times higher (CI_95%_ (IRR) = (1.07, 1.15), *p* ≤ 0.001) during a heat wave than during a control period. In addition to model comparison by AICs, a quantile-quantile plot of the residuals was visually inspected for indications of serious model inadequacies, but none were detected. One hospital was identified as an outlier in this data set and did not fit the model well. This hospital was busier than the other rural hospitals in Southern Ontario.

As the primary predictor variable of interest, HSI was modeled with other variables to test for possible confounding as well. When HSI was modeled in multivariable Poisson analysis, testing for spatial trend polynomial in the hospital location coordinates as confounders, the HSI was a significant predictor of emergency room visits. Since increases in the HSI are related to the presence of a heat wave, it was not reasonable to model the HSI together with the binary heat wave indicator variable. Easting and northing coordinates must be included together as a spatially invariant trend model [35]. When accounting for a spatial trend in the east and north coordinates, a one-unit increase in the HSI was predictive of a 1.004 times higher (CI_95%_ (IRR) = (1.0005,1.007), *p* = 0.024), or 0.4 % increase in, rate of emergency room visits in Southern Ontario. When accounting for the HSI, moving Eastward decreased the rate of emergency room admissions, and moving Northward increased the rate of emergency room admissions within the region.

**Fig. 4:**
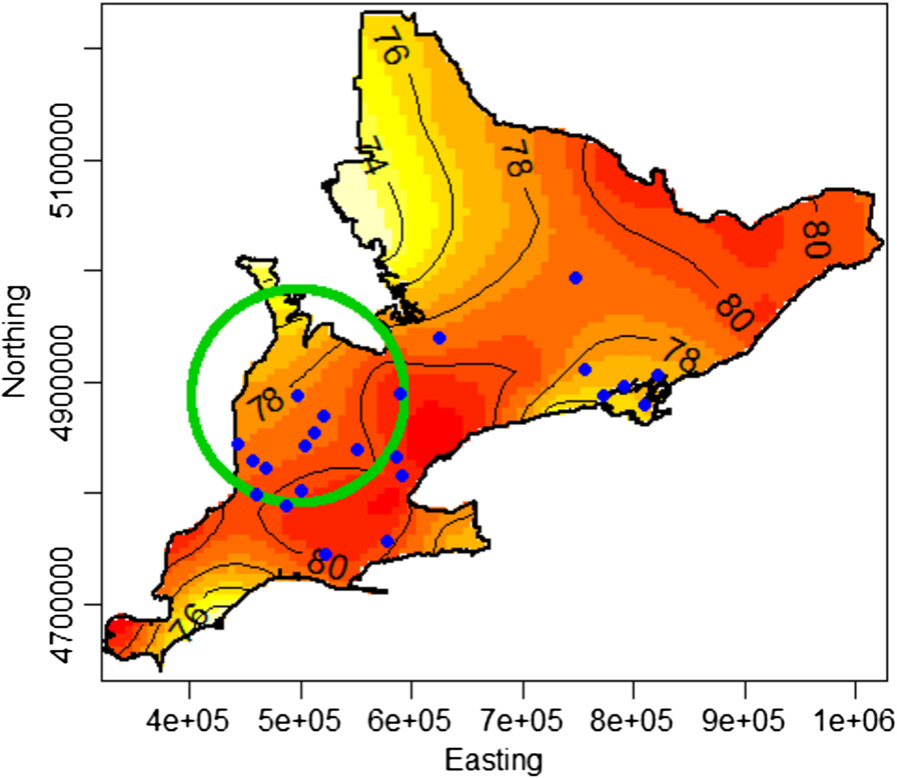
Location of high risk cluster for heat related morbidity during all 3 heat waves. The area enclosed in the circle marks an area of increased risk of heat related morbidity resulting in emergency room visits (IRR = 3.8). The locations of 24 rural community hospitals in Southern Ontario are marked in blue. Axes are in meters

One significant high-risk cluster of emergency room visits to rural hospitals in Southern Ontario was detected using the spatial scan test (*p* ≤ 0.001) during heat waves. The cluster illustrates an increase in the human health impacts of the heat wave, not increased intensity of heat waves in this region. The cluster is located in the southwestern portion of the study region with a radius of 96 kilometers (Fig. 4). Table 2 reports the results from the spatial scan test applied to the study population, i.e. the sample of participating rural community hospitals and their clients. The annual number of cases per 100,000 population is an average of cases during a heat wave at the hospitals in or around the cluster, and accounts for the total population in the study area. The relative risk (RR) within the cluster is 3.8 times higher (*p* ≤ 0.001) than the risk outside the cluster. Although there were some areas outside the cluster that had higher heat stress, there were not enough participating hospitals to see the region was more dramatically affected by climate change. No high or low risk clusters were identified during control periods (*p* > 0.05).

**Table 2:** High risk cluster for increased emergency room visits during heat waves in rural Southern Ontario from 2010–2012

	Inside the Cluster	Total Area
Population	75,280	390,462
Number of cases	6,583	13,838
Annual cases /100,000	8,726	3,537
Observed / Expected	2.47	(ref)
Relative Risk (RR)	3.8	
Size (Radius)	96 km	
P-value	<0.001	

## Discussion

From this study it is apparent that heat stress is a current problem and could be a growing problem for individuals in rural communities in Southern Ontario. Although heat stress is commonly considered a problem for cities, as a result of urban heat islands, it is clear that heat stress is also a problem in small towns and communities. Mapping of heat stress in relation to rural hospitals and Poisson regression models of rural emergency room visits illustrate the increased risk of injury and illness during periods of extreme heat outside of the urban heat island as well. It may also be reasonable to argue that the heat less affects many people in cities, as they are more likely occupied by indoor jobs. In contrast, it is more likely that people residing in rural communities have jobs that require them to spend extended periods of time outside.

The impact of a heat wave significantly increased the rate of admissions to the emergency room. It is likely that the heat wave is widespread across the region and that the effect of a 3-day or longer heat wave is more dramatic than the impact of a change in HSI by a few units. The heat wave indicator is a proxy for increases in the HSI. Therefore joint modeling of the HSI and the heat wave indicator leads to data redundancy issues in multivariable models. On the other hand the HSI alone might be confounded.

The second model indicated that the HSI, although not significant independently, was significant when considering the impact of a linear trend polynomial in easting and northing coordinates of hospital locations as a possible confounder. Since the HSI is spatially varying, the inclusion of an additional trend model in easting and northing coordinates is a point of discussion. Here the spatial trend polynom acted as a confounder, and may have modeled the affect of the Great Lakes or prevailing winds.

It is important to consider the Canadian setting of this paper. The daily maximum temperature of 32 °C is much more dramatically felt in a relatively cool climate, such as Ontario, than it would be in a warmer climate. The impacts of a daily maximum of 32 °C are suggested based on Environment Canada’s extensive research in a Canadian setting, and the possibility that this is a particular threshold for health concerns among the Canadian population.

The impact of heat wave related morbidity in Southern Ontario is substantial in the grand scheme. The increase of 2 hospital emergency room visits per day per rural community hospital suggests an increase during a 3-day heat wave of 6 visits, which at 50 rural hospitals in Southern Ontario amounts to 300 excess admissions to the emergency room per heat wave in rural areas.

The results of the scan test suggest that the southwestern area of the study region, near Lake Huron, is at increased risk of heat related morbidity with an increased number of emergency room visits than its surrounding. This result coincides with being an area previously recognized for its high levels of heat stress during heat waves [31]. The HSI levels in this region do not produce a significant cluster, but the ER visits within the region are significantly higher than in the surrounding area. Bishop-Williams et al. [31] refer to the analysis of clustering of HSI in Southern Ontario in more detail. It is important to note that 48 % or 24 of the 50 eligible hospitals participated in this study. Hospitals in the Eastern area of the study region are disproportionately missing from the dataset, however there are no notable differences between these regions except for the primary language spoken in the community. More French-speaking hospitals are missing as a result of the proximity to French-speaking Quebec in Eastern Ontario, however language would not impact the physiological effects of heat stress on a population.

In rural communities, the catchment area of a hospital is often much larger than that of a hospital in a city. This may impact the results of the study, as the HSI predicted at the hospital may not accurately represent the HSI predicted at the individual’s location at the time of onset of illness. However, based on the maps of heat stress distribution presented in this study, it appears that neighbouring towns experience a similar HSI at any given time. By taking 3-day averages, there may be a smoothing effect, not just in time, but in space as close things are more related than distant things [38]. Therefore, temporal averaging may imply spatial smoothing. This may also have impacted the ability to detect a cluster of high or low risk for emergency room visits.

This study utilized preliminary data without personal identifiers or information about the activity which patients were participating in at the time of injury or onset of illness. It is possible that risky behaviours which are associated with heat but not caused by heat also increased. Such activities as boating which may result in drowning or swimming which may result in slips or injury are likely more common during extreme heat, and thus may inflate the number of hospital visits. This may indicate a possible confounder which may result in overestimating the effect of heat stress on morbidity.

Although an HSI that is generally used in animal populations was applied here to a human population to predict the heat stress distribution across the study region, the maps are predictive of heat stress distribution in humans for Southern Ontario. Barnett et al. [6] tested 5 different HSI estimators with data from 105 cities, and determined that HSI estimators are equally predictive when used correctly. It is therefore best to use the HSI estimator for which there is least missing data. The hourly weather station data retrieved from Environment Canada [5] provided complete recordings for both dry bulb temperature and dew point temperature, as required to estimate the Dairy Cow HSI. Moreover, the Dairy Cow HSI does not use dairy physiology measures to estimate discomfort, but is based solely on environmental factors. Since heat waves have a clear impact on emergency room visits, but HSI was not significant in the univariable regression models due to a confounding spatial trend, further studies are recommended to determine the best meteorological indicator for heat wave impacts on health of rural populations, in order to best reduce or prevent the varied adverse health impacts of heat wave induced heat stress.

The final regression model for heat waves does not fit observations from one of the 24 hospitals well. This particular hospital is much busier than the other hospitals investigated in this study. It is possible, that this hospital is busier because it attracts the population in the neighbouring city catchment during summer months. This hospital is located in an area which is known to be more highly populated during the summer, particularly as travellers pass through toward the Ontario cottage districts. There are no data to confirm this hypothesis; however, it is speculated that a portion of local residents may come to this hospital from the city to avoid traveller congestion along the major summer travel routes. This hospital was not located within the high-risk cluster.

This pilot study illustrated an existing problem of heat-related morbidity in Southern Ontario, for which future surveillance systems may be useful. This study, which is the first of its kind in Southern Ontario, may be used by rural public health units to assess the risk of heat-related morbidity during periods of extreme heat. In many large cities in Southern Ontario, there are cooling centre strategies for heat waves. This study may encourage smaller communities to develop similar strategies. In Southern Ontario, these programs are run at the local level, and may include such things as building cooling centres, water stations along busy pedestrian paths or media alerts. Philadelphia researchers estimate that cooling centres saved 117 lives within the city in 3 years from 1995–1998 [39].

## Conclusion

The rate of increased rural emergency room visits is 1.11 times higher or increased by 11 % during a heat wave compared to a control period. The rate of emergency room admissions increased by 1.004 times, or 0.4 %, per one unit increase in HSI, when accounting for a spatial trend polynom in the easting and northing hospital coordinates. This is a problem across Southern Ontario. In particular in areas such as Southwestern Ontario, that the heat stress maps indicate are at increased risk of heat-related morbidity and where a high-risk cluster was identified there are concerns for the impacts of heat waves on human health.

As heat waves increase in frequency, intensity and duration, the public health burden associated with heat waves will also increase. The results from this study should mobilize rural public health units in Southern Ontario to develop heat stress prevention programs (e.g., by implementing cooling strategies or media alerts during heat waves).
